# Role of the gut microbiota in tumorigenesis and treatment

**DOI:** 10.7150/thno.91700

**Published:** 2024-03-17

**Authors:** Qingya Liu, Yun Yang, Meng Pan, Fan Yang, Yan Yu, Zhiyong Qian

**Affiliations:** 1 Department of Biotherapy, Cancer Center and State Key Laboratory of Biotherapy, West China Hospital, Sichuan University, Chengdu, 610041, China.; 2 Department of Pharmacy, West China Hospital, Sichuan University, Chengdu, 610041, China.

**Keywords:** gut microbiota, dysbiosis, cancer, probiotics, fecal microbiota transplantation (FMT)

## Abstract

The gut microbiota is a crucial component of the intricate microecosystem within the human body that engages in interactions with the host and influences various physiological processes and pathological conditions. In recent years, the association between dysbiosis of the gut microbiota and tumorigenesis has garnered increasing attention, as it is recognized as a hallmark of cancer within the scientific community. However, only a few microorganisms have been identified as potential drivers of tumorigenesis, and enhancing the molecular understanding of this process has substantial scientific importance and clinical relevance for cancer treatment. In this review, we delineate the impact of the gut microbiota on tumorigenesis and treatment in multiple types of cancer while also analyzing the associated molecular mechanisms. Moreover, we discuss the utility of gut microbiota data in cancer diagnosis and patient stratification. We further outline current research on harnessing microorganisms for cancer treatment while also analyzing the prospects and challenges associated with this approach.

## 1. Introduction

The human gut microecosystem is a highly intricate system, and the gut microbiota plays a pivotal role in human health through interactions with the host 1,2. However, cultivating most of the gut microbiota in vitro is challenging, which hinders a comprehensive understanding of these intricate interactions 3,4. The advent of high-throughput sequencing technology (HTST) has propelled the investigation of uncultured microbial communities. As a result, researchers have shifted their focus from merely characterizing the composition of microbial communities to exploring the intricate interplay between these communities and human health, as well as elucidating the underlying mechanisms 5. In terms of genetic constitution, the number of bacteria in the gut microbiota is more than 25 times greater than that in the host, and the gut microbiota consists mainly of bacteria 6. Compared to the human genome, this community can perform a wider range of metabolic functions; however, achieving these metabolic functions through the human genome is difficult 7,8. For this reason, scientists refer to the gut microbiota as the “second genome”.

A substantial body of research has demonstrated a significant correlation between dysbiosis of the gut microbiota and the prevalence of nervous system disorders 9, cancer 10, gastrointestinal ailments 11, cardiovascular conditions 12, and other diseases 13-15. Cancer is a major public health issue and remains one of the leading causes of death worldwide 16. An increasing number of studies indicate that dysbiosis in the gut microbiota is a significant factor in cancer progression; such dysbiosis is now recognized by the scientific community as a hallmark of cancer 17,18. There is compelling evidence that *Fusobacterium nucleatum* and *Bacteroides fragilis* are significantly associated with the pathogenesis of colorectal cancer (CRC) 19,20. It is plausible that *F. nucleatum* and *B. fragilis* may not be the sole potential pathogens. Bullman noted “Every day now there seems to be some new microbe associated with cancer” 21. Although studies have shown that dysbiosis of the gut microbiota is associated with tumorigenesis, further clinical investigations and preclinical studies are warranted to elucidate the underlying mechanisms involved.

The gut microbiota not only plays a role in tumorigenesis but also actively participates in various physiological processes within the human body, thereby making significant contributions to human health 22,23. They serve as signaling molecules implicated in regulating host physiological states, including systemic blood pressure control, modulating the inflammatory response, and preserving the functionality of specific cells 24. Moreover, they facilitate the decomposition of contents within the gastrointestinal tract, where dietary fiber and complex polysaccharides are metabolized into short-chain fatty acids (SCFAs), such as acetate, propionate, and butyrate. Among these SCFAs, butyrate is recognized as a pivotal modulator of the immune response 25. In addition, the gut microbiota also stimulates the development of the host immune system through other means, subsequently influencing overall immune function within the body 26,27. The gut microbiota impacts the immune system, thus contributing to its influence on cancer immunotherapy efficacy. The relationship between cancer immunotherapy and the gut microbiota has garnered significant attention since an article published in 2015 by Science reported that the modulation of the immunosuppressive response is influenced by the gut microbiota 28. In addition to influencing cancer immunotherapy efficacy, the gut microbiota also modulates the efficacy of drugs. Drugs introduced into the gut interact with the resident gut microbiota. These drugs can alter the composition and abundance of the gut microbiota, while the enzymes produced by them can modify drug structure, thereby influencing drug biological activity and toxicity 29,30. Currently, this topic is being explored as a potential target for optimizing drug therapy.

The gut microbiota has been found to be associated with the physiological and pathological status of the host 2,31. Can cancer treatment be optimized through the regulation of the gut microbiota? Among the various factors contributing to patients' unresponsiveness to immune checkpoint inhibitors (ICIs), regulation of the gut microbiota appears to be a readily modifiable aspect through human intervention, with the aim of enhancing patient response to ICIs 32. *B. fragilis* exhibits potential for enhancing the therapeutic efficacy of CTLA-4 blockade 33. Supplementation with *Akkermansia muciniphila* has the potential to augment the efficacy of PD-1 inhibitors 34. Current regulatory approaches and ongoing research on the gut microbiota include manipulating the dietary composition to selectively promote the growth of specific microorganisms 35, administering probiotics 36, performing FMT 37, and targeting the inhibition of specific microorganisms 38. These strategies have the potential to enhance cancer treatment efficacy or mitigate the detrimental effects caused by certain pathogenic microorganisms on the host.

In this review, we focus on the influence of dysbiosis of the gut microbiota on tumorigenesis and treatment response across multiple cancer types while also analyzing the associated molecular mechanisms. Additionally, we briefly explored the use of the gut microbiota as a biomarker for cancer diagnosis and treatment. Subsequently, we elucidate the contemporary approaches employed to regulate the gut microbiota and address their respective prospects and challenges.

## 2. Is the gut microbiota implicated in tumorigenesis?

**Figure 1:**
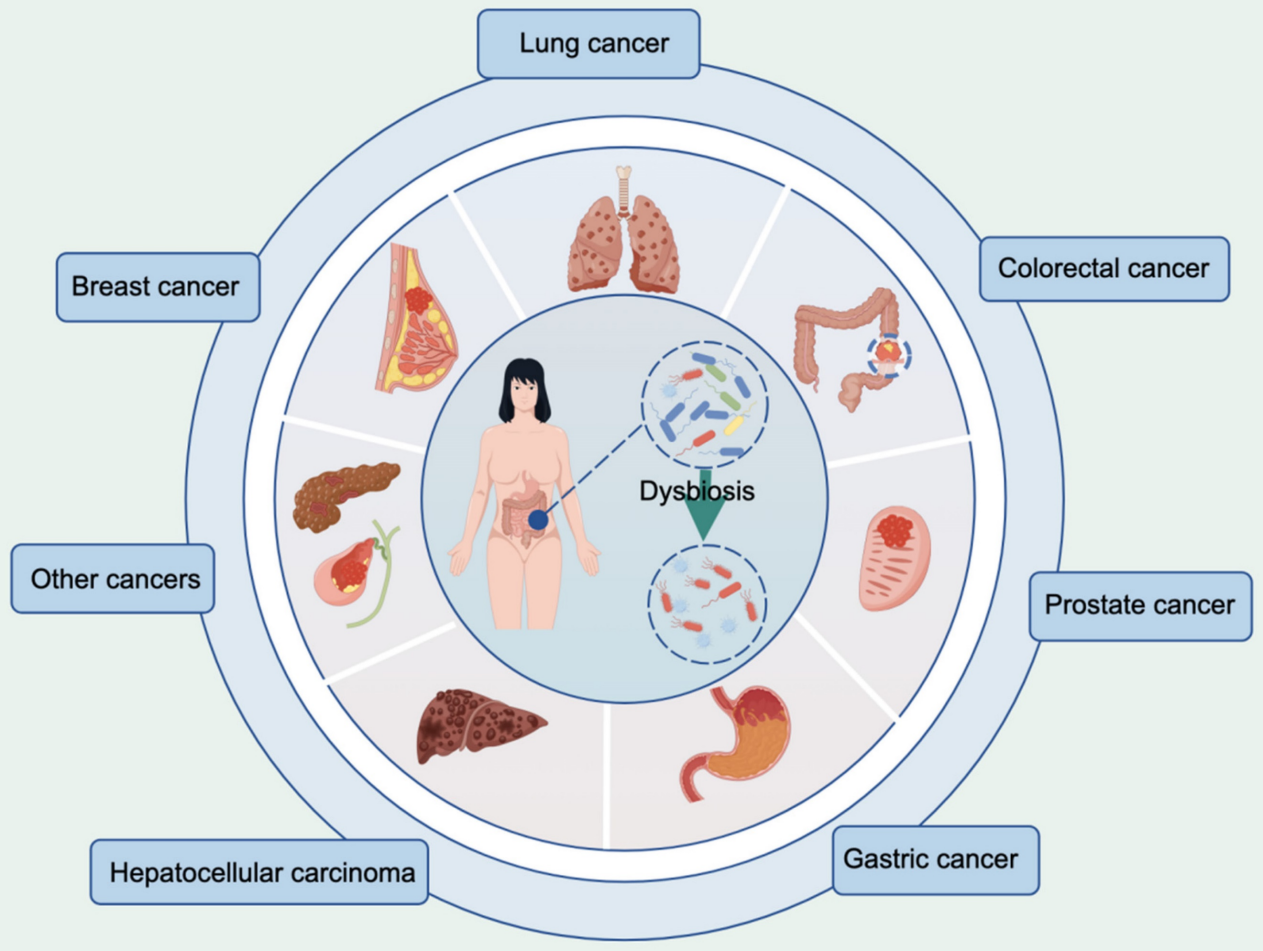
Cancer types associated with dysbiosis of the gut microbiota. This figure was created using Figdraw.

According to the latest global cancer burden figures released by the International Agency for Research on Cancer, breast (11.7%), lung (11.4%), CRC (10.0%), prostate (7.3%), stomach (5.6%) and liver (4.7%) cancers account for the top six new cases of cancer worldwide 39. Substantial experimental and epidemiological evidence strongly supports the influence of the gut microbiota on these types of cancer, as shown in Figure 1. Alterations in the composition of the gut microbiota and associated metabolites have the potential to modulate cellular metabolism and human immune function, thereby establishing a plausible link to cancer 40,41. Despite the increasing interest in this field, there is currently no standardized framework for investigating the correlation between the gut microbiota and cancer incidence, particularly regarding the interpretation of research findings 42,43. Here, we explored the influence of the gut microbiota on these six cancers while analyzing the underlying mechanisms involved.

### 2.1 Breast cancer

The incidence of breast cancer (BC) has surpassed that of lung cancer (LC), making it the leading type of cancer worldwide 39. Recent research has suggested that the gut microbiota may significantly influence BC pathogenesis 17,44. Hou et al. conducted an analysis of the gut microbiota of 267 BC patients and observed that premenopausal individuals with BC exhibited reduced diversity in their gut microbiota and a decreased abundance of probiotics containing tumor suppressor factors and that postmenopausal patients displayed increased levels of pathogenic bacteria. Additionally, they identified 14 microbial markers associated with different menopausal statuses in patients with BC 45. Goedert et al. conducted a comparative analysis of the fecal microbiota of 48 postmenopausal BC patients before treatment and 48 women with normal mammography results. Consistent with the findings of Hou et al., their study also revealed diminished diversity in the gut microbiota among BC patients. Furthermore, they identified higher levels of *Clostridiaceae*, *Faecalibacterium*, and *Ruminococcaceae* as well as lower levels of *Dorea* and *Lachnospiraceae* in BC patients 46.

**Figure 2:**
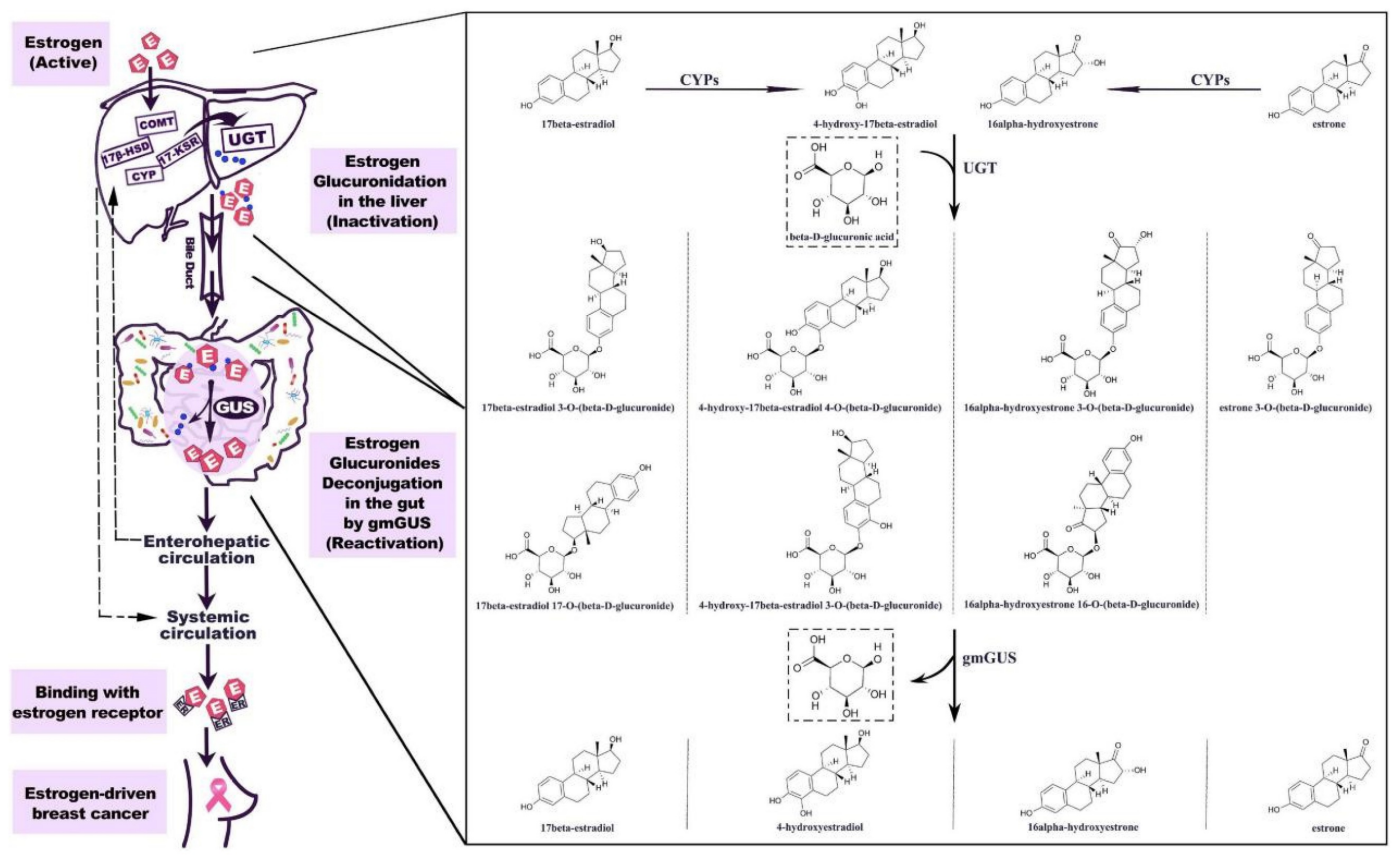
Estrogen metabolism is mediated by gut microbial β-glucuronidase (gmGUS: gut microbial β-glucuronidase). The hepatic metabolism of estrogen is facilitated by a cascade of enzymes. The conjugation of parent estrogens and their phase I metabolites with glucuronic acid can be catalyzed by uridine 5′-diphospho-glucuronosyltransferase (UGT). Estrogen glucuronides are biologically inactive; upon bile excretion, they undergo gastrointestinal transit, during which gmGUS enzymatically hydrolyzes the conjugates to release active estrogens. The reactivated estrogens enter the hepatic circulation and are subsequently reabsorbed into the body. CYP, cytochrome P-450 enzyme. Adapted with permission from 51, Copyright 2021, Sui, Wu and Chen.

Currently, the impact of the gut microbiota on BC can be attributed to its influence on estrogen metabolism 47,48. The incidence of BC is predominantly observed in postmenopausal women; thus, investigations into the relationship between the gut microbiota and BC have primarily concentrated on postmenopausal patients. Postmenopausal women with elevated endogenous estrogen levels are at increased risk of developing BC 49. In 1976, a pioneering study revealed increased excretion of estrogen in the feces of individuals administered ampicillin, suggesting that the gut microbiota enhances estrogen metabolism 47. Gut microbial metabolites, such as β-glucuronidase, influence the levels of nonovarian estrogens through the enterohepatic circulation 48. The human gut microbiota harbors a diverse range of microorganisms, including species such as *Bacteroidetes*, *Firmicutes*, and *Escherichia*, which can encode and produce β-glucuronidases 50,51. Estrogen glucuronides are the primary metabolites of estrogen in liver phase II metabolism. They are excreted into the gut via bile, where β-glucuronidase catalyzes their conversion to free estrogen, which can be absorbed by the gut mucosa and enter the enterohepatic circulation before being distributed to various organs, such as the mammary gland (Figure 2) 51. Among postmenopausal women, an increase in circulating estrogen levels is associated with increased susceptibility to BC. We anticipate that β-glucuronidase-producing microorganisms could be used clinically as biomarkers for predicting BC; alternatively, such microorganisms could be eliminated or β-glucosidase inhibitors could be employed to mitigate the risk of BC development and facilitate treatment 52,53.

Additionally, the modulation of immune responses by the human gut microbiota also influences BC pathology 54. The gut tract serves as the primary immune organ in the human body and exerts a profound influence on human immunity through its pivotal role in promoting the development and maturation of the host immune system, as well as actively participating in regulating overall immune responses 8,55. Neutrophils, a crucial component of the innate immune system, have been implicated in BC progression 56. Clarke et al. demonstrated that the administration of broad-spectrum antibiotics results in the dysregulation of the gut microbiota, thereby impacting neutrophil function in both the serum and bone marrow, consequently compromising innate immune defense 57. The impact of antibiotics on innate immunity suggests that patients receiving immunotherapy in clinical practice should use antibiotics with caution 58,59. Neutrophils are abundant in malignant tumor lesions and play an important role in tumor initiation and progression by generating angiogenic factors, promoting metastasis, and suppressing the immune response to tumors 56,60. A study by Rutkowski et al. provides further evidence of the impact of the gut microbiota on neutrophils. These authors found that *Allobaculum* and *Lactobacillus* were enriched in Toll-like receptor (TLR) 5-deficient mice and that *Bacteroides* was enriched in WT mice. These authors demonstrated that the TLR5-dependent commensal gut microbiota in BC patients can stimulate the systemic upregulation of IL-6, thereby promoting the mobilization of granulocytic myeloid-derived suppressor cells and suppressing tumor immunity, ultimately accelerating the malignant progression of tumors 54. Clinical testing is necessary to ascertain the potential of these microorganisms in BC detection and treatment outcome prediction.

Research findings indicate that breast tissue is not entirely free of bacteria 61. A study by Parida et al. suggested that the promotion of cancer metastasis by microorganisms in breast tissue is associated with specific genera of the gut microbiota 62. They observed that *B. fragilis* was consistently detected in all breast tissue samples, including those from benign and malignant breast cancer patients, as well as in nipple aspirate fluid. The authors fed a cohort of mice harboring *B. fragilis*, which colonizes the gut. As a result, the mice exhibited a significant increase in thickening of the breast duct lining and hyperproliferation of the breast epithelium. The virulence of *B. fragilis* is attributed to the presence of *B. fragilis* toxin 62,63. BC cells exposed to *B. fragilis* toxin for 72 hours retained memory and were capable of initiating cancer development and forming metastatic lesions in mouse models. They also observed the presence of *B. fragilis* in the mammary ducts of mice harboring gut *B. fragilis* infection; however, it remains unclear whether *B. fragilis* translocated internally from the gut to the breast or whether gut-infected mice acquired mammary gland infection through environmental exposure 62.

### 2.2 Lung cancer

The incidence of LC ranks second globally among all types of cancer, yet it remains the primary cause of cancer-related mortality 39. The histological subtypes of LC are categorized as non-small cell lung cancer (NSCLC) and small cell lung cancer (SCLC). Globally, NSCLC accounts for approximately 85% of all lung cancers, with SCLC accounting for the remaining 15% of lung cancers 64. The morphological, etiological, and molecular characteristics of LC have been extensively investigated. In addition to genetic and environmental factors, the gut microbiota plays a pivotal role in the development of LC 65. Qin et al. discovered that, compared with healthy individuals, LC patients exhibit reduced bacterial diversity and that as LC progresses, the levels of SCFAs and anti-inflammatory bacteria decrease; additionally, certain pathogenic bacteria associated with inflammation or tumor promotion were found to be more prevalent among LC patients 66. Zheng et al. recruited 42 early-stage LC patients and 65 healthy individuals to analyze the gut microbiota using 16S ribosomal RNA (rRNA) gene sequencing analysis. They found that *Ruminococcus*, *Enterobacteriaceae*, and *Lachnospiraceae* were highly enriched in the cancer group and that *Faecalibacterium*, *Streptococcus*, *Bifidobacterium*, and *Veillonella* were significantly enriched in the healthy group 67.

The results of the aforementioned clinical studies suggest a potential association between the gut microbiota and the progression of LC. It is widely acknowledged that there are inseparable associations between chronic inflammation and the onset and progression of LC 68. Dysbiosis of the gut microbiota and its metabolites can induce systemic chronic inflammation, thereby contributing to the initiation and progression of LC 69,70. Research by He et al. indicated that antibiotics modulate the gut microbiota to suppress lung inflammation in Treg-deficient mice 71. To investigate the impact of antibiotic-modulated microbiota on suppressing lung inflammation in Treg-deficient SF mice, a treatment regimen involving three different antibiotics was administered to these mice. The results demonstrated that antibiotics reversed the decreases in the relative abundances of the genus *Sutterella* and the family *Mycoplasmataceae* associated with Treg deficiency, thus altering cytokine expression through microbiota-associated metabolites; furthermore, both ampicillin and vancomycin reduced IL-6 levels 71. Sandri's examination of lung tissue revealed that interstitial fibroblasts express IL-6 and contribute to the promotion of cancer 72. The suppression of inflammation in the lungs is achieved through IL-6 blockade. To further validate these findings, IL-6 knockout mice were used to confirm that the deletion of IL-6 confers protection against Treg-induced inflammation 71.

Numerous studies have demonstrated that dysbiosis of the gut microbiota can lead to impaired immune surveillance in lung tissue and create a microenvironment that facilitates the formation of LC cells 73. The impact of the gut microbiota on pulmonary immune function may lie in the activation of gut immunity by the gut microbiota, which leads to the migration of these activated immune cells to the lungs and their involvement in pulmonary immunity. Chemokine-induced homing of lymphocytes plays an important role in this process 74. Congenital lymphocytes in the intestine are closely related to lung homeostasis. The gut microbiota enhances resistance against lung infection by facilitating the recruitment of interleukin-22 (IL-22)-producing group 3 innate lymphoid cells (IL-22^+^ILC3) into the lungs of neonatal mice 75. The interaction between the gut microbiota and intestinal dendritic cells (DCs) (CD103^+^CD11b^+^DCs) induces the upregulation of CCR4-related homing receptors in gut IL-22^+^ILC3s, facilitating the selective migration of gut IL-22^+^ILC3s to the lungs. CCR4 is a chemokine receptor that is commonly identified as a key mediator in the trafficking of T cells and Treg cells to the lungs 76. The chemokine CCL17, which is expressed in lung epithelial cells, activates the CCR4 receptor, thereby facilitating the recruitment of IL-22^+^ILC3s into the lungs of neonatal mice. Elevated levels of IL-22 within the lung environment can impede pathogen proliferation 75. Disruption of commensal bacteria interrupts the migratory program of IL-22^+^ILC3s, impairing their ability to traffic to the lungs and rendering newborn mice more susceptible to pneumonia.

### 2.3 Colorectal cancer

**Figure 3:**
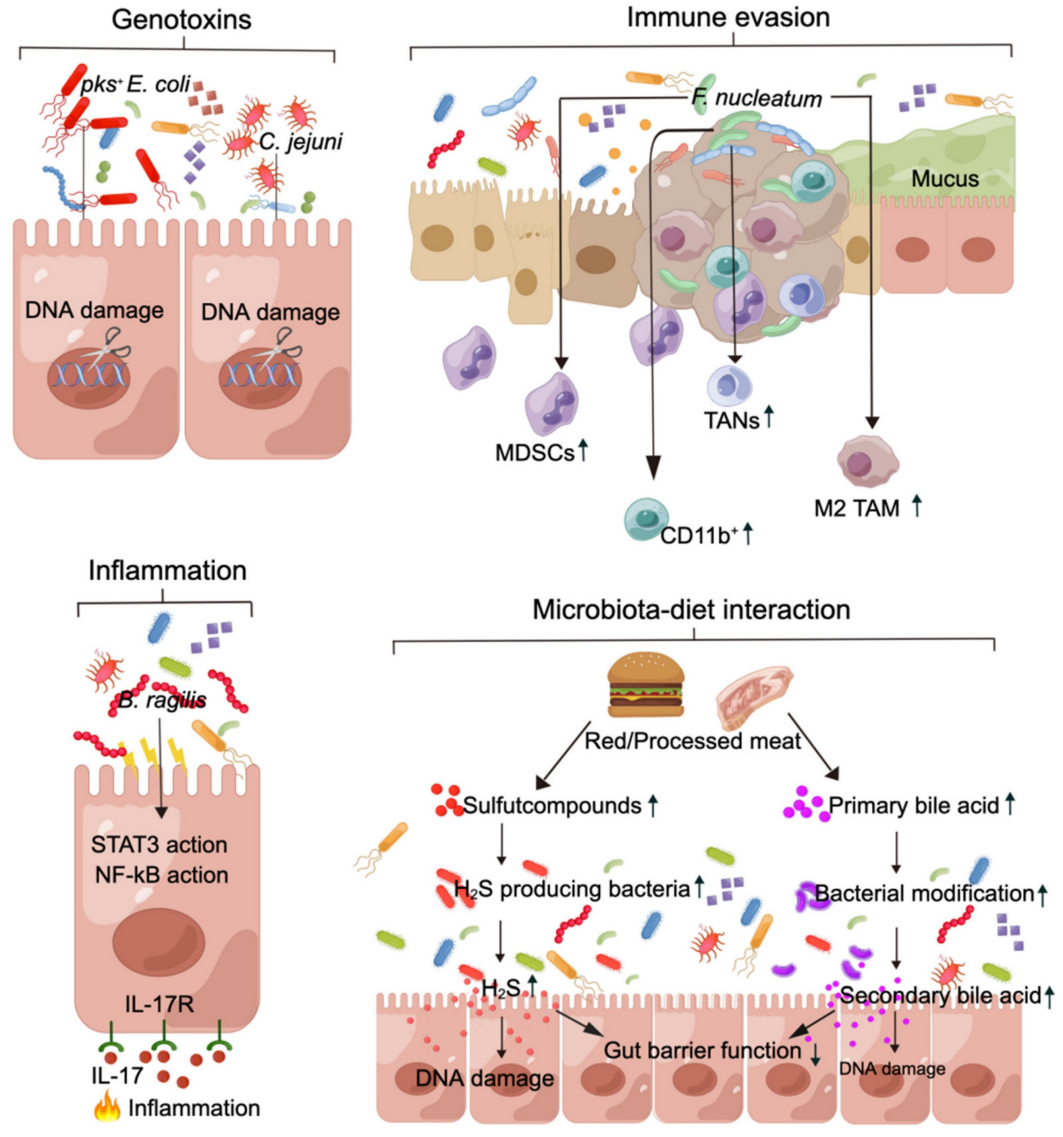
Gut microbiota dysbiosis contributes to the development of CRC through a diverse range of molecular mechanisms. (A) pks^+^ *E. coli* and *C. jejuni* produce genotoxins, which induce DNA damage and increase the frequency of gene mutations, thus contributing to CRC. (B) *F. nucleatum* leads to the expansion of MDSCs, CD11b^+^ cells, M2 TAMs, and TANs. These cells play a crucial role in suppressing antitumor immunity. (C) *B. fragilis* triggers a procarcinogenic, multistep inflammatory cascade involving the IL-17R, NF-kB, and STAT3 signaling pathways in colonic epithelial cells. (D) Red/processed meat can potentially modify the structure and function of the microbiota, leading to increased production of H_2_S and secondary bile acids by microorganisms. These alterations can result in damage to gut barrier function and DNA, thereby elevating the risk of CRC. MDSCs: myeloid-derived suppressor cells; TANs: tumor-associated neutrophils; M2 TAMs: M2-like tumor-associated macrophages; H_2_S: hydrogen sulfide. This figure was created using Figdraw.

CRC is the fourth most common cancer diagnosed worldwide, while it is the third most common cancer 39. A distinctive characteristic of CRC is its close association with the gut microbiota, which constitutes an integral component of the tumor microenvironment 20,77. The gut microbiota in patients with CRC has been extensively investigated, making it arguably the most exemplary illustration of the role played by the gut microbiota in cancer 78. The initial evidence supporting the involvement of the gut microbiota in CRC emerged in 1975 when both germ-free and conventional mice were administered 1,2-dimethylhydrazine. A significantly greater percentage (93%) of conventional mice developed CRC than did germ-free mice (21%) 79. Subsequent studies have demonstrated that specific strains of the gut microbiota, such as *Enterococcus*, *Bacteroidetes*, and *Clostridium*, may contribute to the development of CRC by enhancing crypt lesions induced by 1,2-dimethylhydrazine 80. However, FMT from CRC patients into germ-free mice promoted gut cell proliferation and facilitated the progression of azoxymethane-induced crypt lesions to CRC 81. Genomic sequencing of fecal samples from CRC patients across different regions revealed several core pathogenic species. Notably, these strains, which include *F. nucleatum*, *Parvimonas micra*, *Peptostreptococcus stomatis*, *Peptostreptococcus anaerobius*, *Porphyromonas asaccharolytica*, *Solobacterium moorei* and *Prevotella intermedia*, are also enriched in the oral cavity 82,83. The relationship between the gut microbiota and CRC has been elucidated, encompassing the factors depicted in Figure 3.

Genotoxins. Genetic alterations in the activation of oncogenes and/or inactivation of tumor suppressor genes, which are mediated by toxins produced by the gut microbiota, contribute to tumorigenesis 84,85. For example, colibactin is produced by pks^+^ *Escherichia coli* strains 86, and the cytolethal distending toxin is produced by *Campylobacter jejuni* 85. These toxins induce double-stranded DNA breaks in host cells, triggering a signaling cascade of DNA damage that results in persistent mitosis, chromosomal aberrations, and an increased frequency of gene mutations 86-88. Cao et al. showed that gene toxins within the gut microbiota continuously induce DNA damage in host epithelial cells synergistically with chronic inflammation and other environmental factors within the gut microenvironment, ultimately facilitating the initiation and progression of CRC 87.

Immune evasion. Gut pathogenic bacteria have been shown to promote an immunosuppressive tumor microenvironment that facilitates the growth of CRC, with a particular emphasis on the role of *F. nucleatum* in promoting immune evasion in CRC 89,90. Research findings indicate that an *F. nucleatum* inhibitor protein effectively suppresses human T-cell activity by impeding the G1 phase of the cell cycle, thereby fostering an immunosuppressive microenvironment conducive to tumor cell proliferation 90. Additionally, *F. nucleatum* modulates the tumor immune microenvironment and leads to the expansion of myeloid-derived suppressor cells (MDSCs), CD11b^+^ cells, M2-like tumor-associated macrophages (M2 TAMs), and tumor-associated neutrophils (TANs). These cells play crucial roles in suppressing antitumor immunity and promoting tumor progression 91. Jiang et al. demonstrated that succinic acid derived from *F. nucleatum* inhibits the cGAS-interferon-β pathway, thereby attenuating the antitumor response by restricting CD8^+^ T-cell trafficking to the tumor microenvironment in vivo 92.

Inflammation. Gut microbiota dysbiosis is strongly linked to inflammation of the gastrointestinal tract and plays a crucial role in the initiation of colitis-associated CRC 87,93. The inflammation induced by gut pathogenic bacteria often involves the activation of the IL-17, NF-κB, and pattern recognition receptor (PRR) signaling pathways, as well as disruption of gut barrier function 94,95. These interconnected cascades collectively contribute to a proinflammatory phenotype. Chung et al. demonstrated that the induction of inflammation by enterotoxigenic *B. fragilis* begins with the disruption of gut barrier function and the subsequent activation of STAT3 and NF-κB signaling in IL17Rexpressing colonic epithelial cells in a cascade of inflammation-related responses 95. Thus, myeloid cell-dependent distal colon tumorigenesis is triggered by myeloid cells.

Diet. Studies indicate that the initiation of CRC is associated with dietary constituents, such as the consumption of red meat and processed meat 96,97. Hydrogen sulfide (H_2_S) is produced in the gut by sulfur-reducing bacteria from inorganic sulfur, which is commonly used as a preservative in processed meat, or by fermentative bacteria that metabolize organic sulfur compounds found in animal products such as red meat 96. The microbiota metabolizes these meats to generate nitroso compounds, H_2_S, and other procarcinogens, thereby contributing to the initiation of CRC 98,99. In a cohort of elderly men, there was an association between increased dietary intake of organic sulfur and an increase in the fecal abundance of H_2_S-producing Clostridium clostridioforme 100. In addition, fiber and resistant starch are decomposed by the gut microbiota to produce SCFAs. A reduction in the intake of these substances leads to a decrease in SCFA levels, which has been extensively demonstrated in numerous studies to inhibit the development of CRC 101,102. Elevated levels of secondary bile acids, such as deoxycholic acid, in individuals adhering to a high-fat diet have been linked to increased susceptibility to CRC 96,103.

### 2.4 Prostate cancer

The incidence of prostate cancer (PCa), a prevalent malignancy that poses a significant threat to men's health, ranks fourth globally among newly diagnosed cancers 39, and age, race, and family history are the main risk factors 104. Diet and physical activity also play important roles in the development and progression of PCa, particularly in relation to ethnicity, at different incidence rates 105. Additionally, an increasing body of research over the past decade has demonstrated the significant role that the gut microbiota plays in the occurrence and development of PCa 106,107. Liss et al. conducted an analysis of the gut microbiota in 133 U.S. men who underwent prostate biopsy; they performed 16S rRNA amplicon sequencing on 105 samples (64 with cancer and 41 without cancer) 108. These findings revealed enrichment of Bacteroides and Streptococcus species among PCa patients. Furthermore, a subsequent metagenomic analysis demonstrated significant alterations in the arginine and folate pathways within the gut microbiota. Consequently, the authors propose that the gut microbiota may influence the risk of developing PCa. Matsushita et al. conducted an analysis of 152 Japanese men who underwent prostate biopsies; 96 had cancer, and 56 did not 109. The results showed that the relative abundances of Rikenellaceae, Alistipes, and Lachnospira significantly increased in the high-risk group (men with grade 2 or above PCa) and the negative + low-risk group (men with biopsy negative or grade 1 PCa).

The results of the aforementioned studies provide preliminary evidence suggesting a correlation between the gut microbiota and PCa incidence, but the impact of the gut microbiota on PCa incidence is still under investigation. The etiology of PCa primarily involves excessive androgen production, and androgen deprivation therapy (ADT) is commonly used for treatment 110. Although initially effective, this treatment can lead to a transition in patients' condition from hormone-sensitive prostate cancer to castration-resistant prostate cancer (CRPCa) as therapy progresses 111. Pernigoni et al. reported that the gut microbiota contributes to the development of CRPCa by providing an alternative source of androgens 112. Surgical castration (CT) was performed on mice, which subsequently progressed to the castration-sensitive phase (CS), characterized by a rapid decrease in prostate cancer tumor volume. Subsequently, the mice progressed to the castration-resistant phase (CR), which was characterized by a gradual increase in tumor volume. The subsequent depletion of the gut microbiota in CT mice through antibiotic treatment resulted in a significant reduction in tumor volume. 16S rDNA sequencing analysis revealed a significant increase in the abundance of *Ruminococcus gnavus* and *Bacteroides acidifaciens* in CR mice. Furthermore, it has been confirmed that *Ruminococcus sp.* DSM_100440 is capable of metabolizing pregnenolone and hydroxypregnenolone into androgens such as dehydroepiandrosterone and testosterone in a mouse model.

### 2.5 Gastric cancer

Gastric cancer (GC) is the fifth most prevalent and fourth most deadly malignancy worldwide, making it one of the leading causes of death globally 39. The gastrointestinal microbiota plays a crucial role in the occurrence and progression of GC 113. *Helicobacter pylori* infection significantly increases the risk of GC 114; however, it cannot solely account for all cases of GC 115. The advancement of high-throughput sequencing technology has facilitated an increasing number of studies investigating the association between the gut microbiota and GC 116,117. Li et al. analyzed the gut microbiota of 130 patients with gastrointestinal tumors and 147 healthy controls and found significant differences in the composition and abundance of the gut microbiota between the two groups 118. Zhou et al. sequenced 16S rRNA target genes from tumor tissue and fecal samples of 1043 participants from 10 hospitals. *Streptococcus anginosus* and *Streptococcus constellatus* were enriched in both the tumor tissue and feces of GC patients, with a stronger enrichment signal observed in the feces than in the tissue samples 119.

With the advancement of related research, the underlying mechanism by which *H. pylori* contributes to GC pathogenesis has been progressively elucidated. Studies have shown that *H. pylori* can induce the production of ROS and that excessive ROS can lead to oxidative stress, resulting in DNA damage and thus the formation of tumor precursors 120. The proliferation and apoptosis of gastric epithelial cells are normal physiological phenomena. *H. pylori* infection leads to increased apoptosis and proliferation of gastric epithelial cells, but proliferation still dominates, which may be one of the causes of GC 121. *H. pylori* induces a strong inflammatory response, which may play an important role in the progression from chronic inflammation to gastric malignancy 122. An important characteristic of *H. pylori* infection is the rapid recruitment of regulatory T cells and myeloid cells (including dendritic cells, neutrophils, and M1 macrophages) to the stomach for the secretion of a series of cytokines (such as IFN-γ, IL-17, and IL-21), which collectively establish an immunosuppressive microenvironment prior to the development of gastric epithelial cell malignancy 123.

A study conducted by Zhou et al. demonstrated the enrichment of *S. anginosus* in both tumor tissue and the intestinal microbiota among patients with GC 119. It is an emerging pathogen with previously unrecognized pathogenic potential that has recently garnered increased amounts of attention in the scientific community. Asam et al. demonstrated that streptolysin, produced by *S. anginosus*, functions as a broad-spectrum hemolysin and cytolysin capable of facilitating bacterial translocation across the epithelial barrier, inducing tissue damage, and destroying neutrophils and macrophages to evade host immune escape 124. Sasaki et al. isolated and purified an antigen from the bacterial supernatant of *S. anginosus* 125. This antigen can stimulate macrophages to synthesize nitric oxide (NO), leading to intracellular oxidative stress and lipid peroxidation, ultimately resulting in DNA damage and subsequent tumorigenesis 126.

### 2.6 Hepatocellular carcinoma

Hepatocellular carcinoma (HCC) is the fourth leading cause of cancer mortality worldwide, accounting for approximately 90% of liver cancer cases and posing a significant global healthcare challenge 18,127. HCC predominantly arises in patients with underlying chronic liver disease and is propelled by an intricate interplay of hepatic injury, inflammation, and regeneration that typically spans several decades 127,128. Emerging evidence strongly supports the pivotal role of alterations in the gut barrier and the composition of the gut microbiota in driving the progression of chronic liver disease and facilitating HCC development 129. Research findings indicate that a significant increase in lipopolysaccharide (LPS) levels in HCC patients is accompanied by gut barrier disruption 129,130. Impairment of the gut barrier may contribute to the excessive production of LPS by the gut microbiota into the portal vein and liver, further triggering HCC 131. Additionally, Ren et al. conducted a comprehensive analysis by collecting 419 samples, revealing the enrichment of *Actinobacteria*, *Gemmiger*, and *Parabacteroides* in early HCC 132. Ni et al. demonstrated that patients with primary HCC exhibited an increase in proinflammatory bacteria within their fecal microbiota, and the degree of dysbiosis in the gut microbiota was significantly greater than that in healthy controls 133. These research findings provide evidence supporting a potential association between HCC and the gut barrier, as well as the composition of the gut microbiota, thereby contributing to the elucidation of this relationship through scientific investigation.

The hepatic artery in the abdominal cavity and the portal vein delivered by the gut and spleen constitute the dual blood supply of the liver, with 75% of its blood being supplied by the portal vein 129. The blood from the gut portal vein not only contains nutrients but also carries substances such as LPS and peptidoglycan from the gut microbiota 134,135. Usually, these substances are present in minimal quantities and can be efficiently cleared by Kupffer cells in the liver without eliciting any detrimental effects on the host 135. The maintenance of these physiological conditions relies on the integrity of the gut barrier.

**Figure 4:**
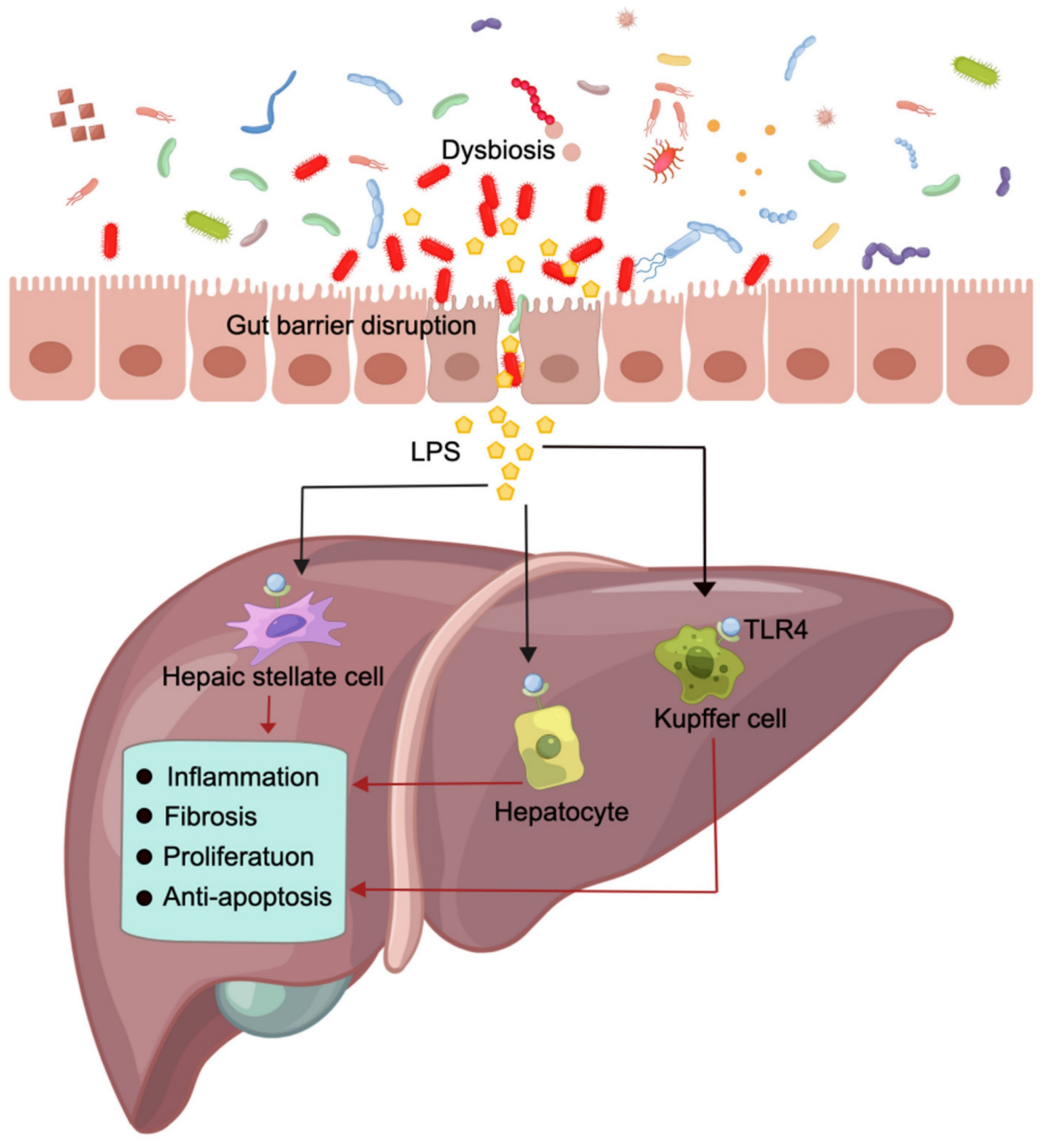
Contribution of the gut microbiota to HCC and the underlying mechanisms involved Dysbiosis of the gut microbiota and impairment of the gut barrier result in the translocation of LPS from the gut lumen to the bloodstream, leading to increased hepatic exposure to LPS. This promotes hepatic inflammation, fibrosis, proliferation and the activation of antiapoptotic signals. This figure was created using Figdraw.

Dysbiosis of the gut microbiota can reduce the diversity and abundance of probiotics while promoting the growth of pathogenic bacteria 136,137. Consequently, this disrupts the integrity of the gut barrier, facilitates bacterial translocation, and allows for substantial entry of LPS into the portal vein and liver. The impairment of the gut barrier promotes hepatic inflammation, fibrosis, proliferation and the activation of antiapoptotic signals by activating LPS and its receptor TLR. This process thereby facilitates the development of HCC (Figure 4) 131,138. Administering dextran sulfate sodium disrupts the gut barrier, which not only results in increased systemic LPS levels and liver fibrosis but also promotes HCC formation in mice 139. However, inhibiting TLR4 signaling suppressed liver inflammation, fibrosis, and HCC formation in both mice and rats 131. Experiments conducted on TLR4 chimeric mice have demonstrated that the expression of TLR4 on liverresident cells, including hepatocytes and Kupffer cells, is accountable for promoting fibrogenesis and HCC 140. Activation of the LPS-TLR4 signaling pathway in Kupffer cells has been shown to induce TNF-dependent and IL-6-dependent compensatory hepatocyte proliferation while also reducing oxidative stress and apoptosis 141. Additionally, the activation of TLR4 in HCC cell lines induced by LPS enhances the invasive potential of these cells and induces epithelial-mesenchymal transition 142,143.

The involvement of the gut microbiota in the pathogenesis of various types of cancer, including BC 46, LC 65 and CRC 77, has been confirmed in clinical and preclinical studies. However, the clinical lineups in some studies had a limited sample size 46,67. Moreover, the incidence rates of the same cancer also vary across different regions worldwide. For instance, in Southern Europe, the incidence rate of CRC is 25.3 per 100,000 males and 16.4 per 100,000 females, whereas in Middle Africa, it is only 2.9 per 100,000 males and 2.3 per 100,000 females 39. It is necessary to include larger and more regional populations to further refine and validate the association between the gut microbiota and corresponding cancers. The ultimate goal is to provide a new method based on the gut microbiota for early cancer screening, enabling its prompt diagnosis and treatment 132.

## 3. Questioning the impact of the gut microbiota on cancer treatment

**Figure 5:**
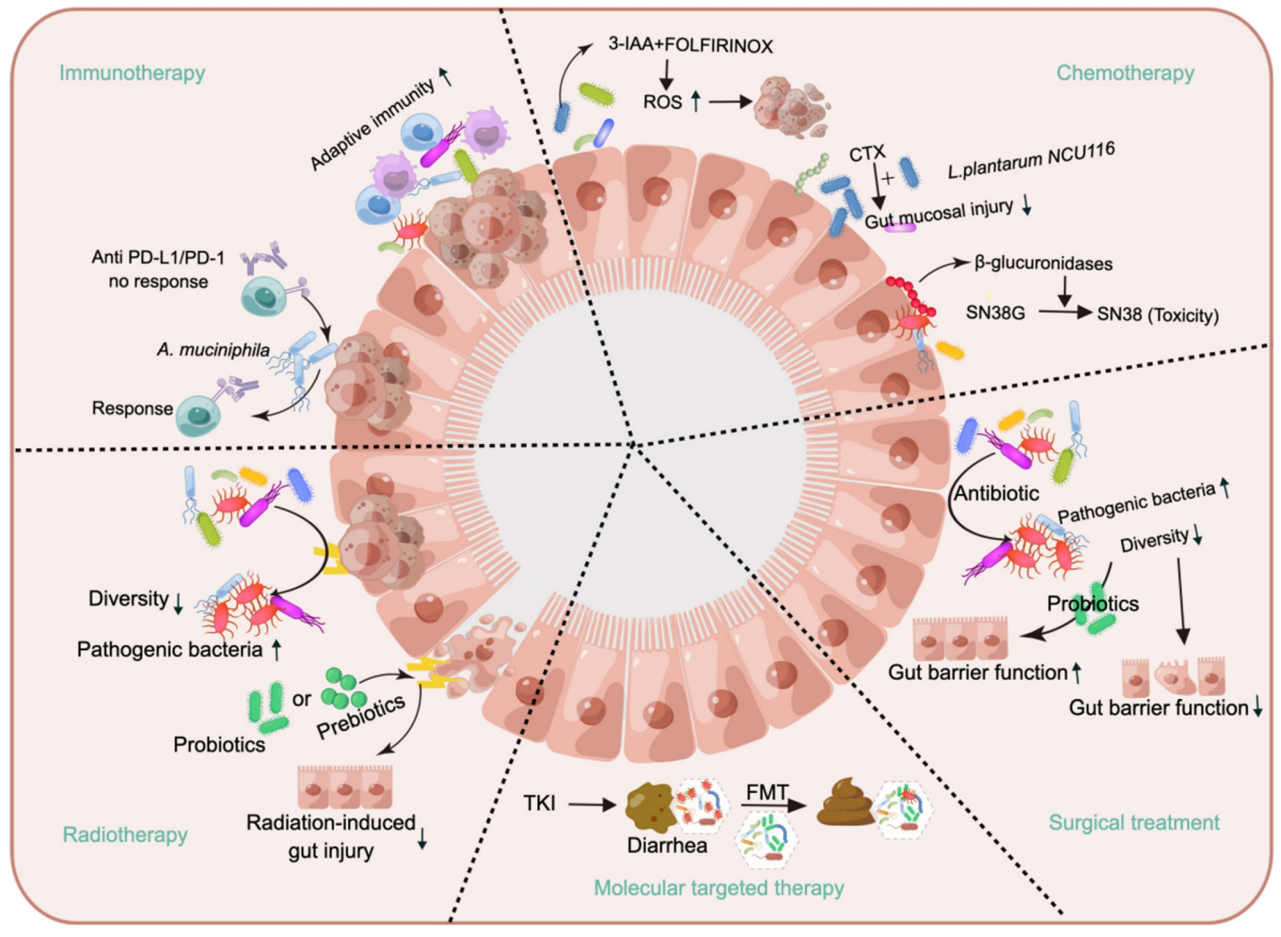
This article provides an overview of gut microbiota-cancer therapy interactions. The gut microbiota induces adaptive immunity, and *A. muciniphila* reverses the nonresponse to PD-1/PD-L inhibitors. The metabolite 3-IAA, produced by the gut microbiota, enhances the efficacy of chemotherapy. Supplementation with *Lactobacillus plantarum NCU116* can reduce the damage caused by CTX to the gut mucosa. However, β-glucuronidases produced by the gut microbiota can convert SN-38G into SN-38, which is toxic to the gut. Radiation therapy can result in a reduction in the diversity of the gut microbiota and an increase in the abundance of pathogenic bacteria, whereas supplementation with probiotics and prebiotics exerts a protective effect against radiation-induced damage. The use of FMT is indicated for the treatment of diarrhea resulting from TKI therapy. The administration of preoperative antibiotics may result in a reduction in gut microbial diversity and the proliferation of pathogenic bacteria, which can compromise gut barrier function. Conversely, probiotic supplementation has been shown to enhance gut barrier function. This figure was created using Figdraw.

While some gut microbiota may promote the initiation and progression of cancer, not all gut microbiota are harmful; in fact, certain types of gut microbiota can be beneficial for cancer treatment 144,145. Research on the use of the gut microbiota for treating cancer has focused primarily on enhancing human immunity 145,146, but the benefits of the gut microbiota are not limited to immune enhancement; it can also improve chemotherapeutic efficacy and mitigate adverse effects 147,148, as shown in Figure 5.

### 3.1 Immunotherapy

The host acquires microbiota from the environment at birth; this microbiota interacts with the immune system during the first 2-3 years of life and subsequently establishes a stable microbiota 149. This community promotes both innate and adaptive immunity at multiple levels 150. The evolution of the innate and adaptive immune systems leads not only to the elimination of specific pathogens but also to the shaping of the composition of the commensal gut microbiota 149. The immune system also undergoes its own progression 26.

Considering the history of microorganisms as anticancer tools, they were first recognized for having such effects in the 19th century. Busch observed tumor regression in cancer patients after infection with erysipelas, and Fehleisen identified *Streptococcus pyogenes* as the causative agent of erysipelas infection. Afterward, Coley developed the first cancer immunotherapy drug (“Coley toxin”) based on heat-killed bacteria 151. Over 1000 patients, many or most of whom had sarcomas, exhibited degenerative changes and cures. However, this “medicine” gradually failed because it was not administered following a scientific protocol and could not consistently achieve reproducible results 152. The advent and advancement of radiotherapy, chemotherapy, and targeted therapy have long overshadowed immunotherapy as effective methods for cancer treatment 153,154. After nearly a century of effort, with the emergence of immunosuppressants, initial results have been achieved in the application of immunotherapy in cancer treatment. The Food and Drug Administration (FDA) has approved several immune drugs, either alone or in combination with other drugs, for the treatment of various malignancies, e.g., ipilimumab 155, nivolumab 156, and imiquimod 157. However, clinical studies have shown that not all ICIs are effective for every patient 28,158. Since the publication of an article in Science in 2015 demonstrating that the gut microbiota can reverse nonresponse to immunosuppression, there has been renewed interest in exploring the influence of the gut microbiota on tumor immunotherapy 28.

The adaptive immune response induced by the gut microbiota primarily occurs through interactions between pathogen-associated molecular patterns (PAMPs) and PRRs 159,160. Local immune responses are initiated when PRRs (e.g., TLRs) recognize PAMPs from the gut microbiota 161. Microorganisms or their metabolites (e.g., SCFAs) can serve as inducers of local immunity. During this process, SCFAs activate the plasma cell production of IgA, which hinders bacterial adhesion, aggregation, and invasion while also directly affecting bacterial virulence 26. In addition, PAMPs induce DC maturation. Mature DCs then migrate to mesenteric lymph nodes where they interact with naive T cells, facilitating their development into CD4^+^ T cells. The stimulation of CD8^+^ T cells is also directly induced by DCs, and activated T cells play a crucial role in maintaining the stability of the gut environment and preventing gut infections 26.

The survival rate of patients with epithelial tumors who did not receive antibiotic treatment during anti-PD1/PD-L1 therapy was significantly greater than that of patients who received antibiotics 162,163. A comparison of the gut microbiota between PD-1 inhibitor responders and nonresponders revealed significant differences, particularly in terms of the greater diversity observed among responders 164. Additionally, the abundance of *A. muciniphila* in fecal samples from patients who exhibited a positive response to PD-1 inhibitors was significantly greater than that in nonresponders 162. Oral administration of *A. muciniphila* can ameliorate patient responsiveness to PD-1 inhibitors through the promotion of immune cell infiltration into tumors through supplementation with *A. muciniphila*. Specifically, CR9^+^, CXCR3^+^, and CD4^+^ T cells are recruited to the tumor microenvironment, where they restore the efficacy of PD-L1 inhibitors 165.

Chimeric antigen receptor (CAR) T-cell therapy, a state-of-the-art immunotherapy, represents a novel therapeutic avenue for patients with refractory and recurrent B-cell leukemia or lymphoma. However, the efficacy of this treatment remains heterogeneous, with only 40% of patients achieving complete and durable remission at best, thereby impeding its widespread clinical application 166,167. Stein-Thoeringer et al. demonstrated that exposure to broad-spectrum, high-risk antibiotics (such as meropenem, piperacillin-tazobactam or cefepime) within 3 weeks prior to CAR-T-cell therapy is associated with worse progression-free survival and overall survival 59. Next, they analyzed the gut microbiota and found that *Bacteroides*, *Ruminococcus*, *Eubacterium* and *Akkermansia* are the most important genera for determining CAR-T-cell responsiveness. Additionally, *Akkermansia* was shown to be associated with preinfusion peripheral T-cell counts in these patients. Luu et al. reported that the frequency of IFN-γ^+^TNF-α^+^CD8^+^ T cells within cytotoxic T lymphocytes significantly increased after treatment with supernatants derived from *Megasphaera massiliensis*, and they demonstrated that this increase was caused by SCFAs in the supernatant 168. Further studies have shown that treatment with butyrate or pentanoate can enhance the expression of CD25 and the production of TNF-α and IFN-γ in receptor tyrosine kinase-like orphan receptor 1 CAR-T cells upon stimulation, thereby enhancing the antitumor efficacy of CAR-T cells.

CpG-oligodeoxynucleotide (CpG-ODN) is a TLR9 agonist with an immunoactivating effect that can directly induce the activation and maturation of plasmacytoid dendritic cells as an adjuvant for tumor immunotherapy 169. Studies have shown that CpG-ODNs activate the immune response to tumor cells by inducing the secretion of proinflammatory cytokines such as TNF-α and IL-12 from myeloid cells 170. Iida et al. reported that in this proinflammatory microenvironment, antigen-specific T-cell activation occurs, resulting in the effective clearance of most conventional mouse tumors. However, tumor-infiltrating myeloid cells in germ-free mice fail to produce proinflammatory agents that respond to CpG-ODNs, leading to diminished therapeutic efficacy of CpG-ODN therapy 171. Additionally, they investigated the association between the gut microbiota and CpG-ODN efficacy and revealed a positive correlation between *Ruminococcus obeum* and *Alistipes* and TNF-α secretion, with *Lactobacillus sp.* exhibiting a negative correlation. Subsequent administration of *Alistipes* following antibiotic treatment restored CpG-ODN-induced TNF-α secretion, whereas oral administration of *Lactobacillus sp.* reduced TNF-α secretion.

### 3.2 Chemotherapy

Currently, chemotherapy is the conventional therapeutic approach for treating pancreatic ductal adenocarcinoma; however, approximately 50% of patients do not respond to this therapeutic approach 172,173. Genetic alterations in patients cannot explain the difference between responsive and nonresponsive patients to chemotherapy 174,175. Emerging evidence highlights the pivotal role of the gut microbiota in determining the response to chemotherapy 173. Tintelnot et al. reported that the microbiota-derived tryptophan metabolite indole-3-acetic acid (3-IAA) is enriched in patients who respond to chemotherapy 173. Furthermore, they demonstrated that the efficacy of 3-IAA and chemotherapy is mediated by neutrophil-derived myeloperoxidase. In conjunction with chemical treatment, myeloperoxidase oxidizes 3-IAA, which leads to the downregulation of the enzymes glutathione peroxidase 3 and glutathione peroxidase 7, which are responsible for degrading reactive oxygen species (ROS). The accumulation of ROS and the downregulation of autophagy compromise the metabolic fitness of cancer cells, ultimately impeding their proliferation. The gut microbiota not only enhances the effectiveness of chemical drugs but also alleviates their adverse effects 148,170. When chemotherapy drugs act on rapidly proliferating tumor cells, gut mucosal cells are also affected by their high proliferation rate, resulting in disruption of the gut barrier 176. Cyclophosphamide (CTX) is a commonly used chemical drug that is widely employed in the treatment of patients with solid tumors and hematological malignancies 177. However, it is known to induce acute gut mucosal injury 178. Oral administration of *Lactobacillus plantarum NCU116* has significant efficacy in ameliorating CTX-mediated gut mucosal injury and improving gut metabolism and the gut microbiota 179.

The gut microbiota exerts both beneficial and detrimental effects on chemotherapy outcomes 179,180. Relevant studies have demonstrated that irinotecan, a chemical drug frequently used for treating CRC, can induce severe diarrhea due to the presence of bacterial β-glucuronidase in the gut 180. Carboxylesterases, which are present in various tissues, catalyze the conversion of CPT-11 into SN-38, thereby killing cancer cells. Moreover, SN-38 can be inactivated in the liver by the uridine diphosphoglucuronosyltransferase 1A1, resulting in the formation of SN-38G. Subsequently, SN-38G is excreted into the gut via bile. Although SN-38G does not exhibit toxicity toward the gut mucosa, the enzymatic activity of β-glucuronidases produced by the gut microbiota leads to the metabolic conversion of SN-38G into SN-38, which damages the gut mucosa 180,181.

### 3.3 Radiotherapy

Radiotherapy is an important method for treating tumors because it induces DNA damage in both tumor cells and normal cells through indirect energy transfer, which involves the production of reactive oxygen and nitrogen 182. Gastrointestinal cells exhibit rapid turnover and are highly susceptible to radiation, making them the primary target of injury during radiotherapy and significantly impacting patient quality of life 183. Numerous studies have demonstrated the crucial role of the gut microbiota in regulating the physiological and pathological states of the host 1,2, suggesting its potential involvement in radiation-induced damage 184. Ferreira et al. conducted a cohort study to investigate the relationship between the gut microbiota and radiation-induced enteropathy. They found that patients with radiation enteropathy had a reduced diversity of gut microbiota, as well as a significantly greater abundance of *Clostridium IV*, *Roseburia*, and *Phascolarctobacterium* 185.

Guo et al. reported that only a small percentage of mice were able to survive a high dose of radiation. Subsequent research revealed enrichment of *Lachnospiraceae* and *Enterococcaceae*, which can mitigate radiation-induced gastrointestinal damage, in these elite survivors. Through nontargeted metabolomics research, they discovered that downstream metabolites of the gut microbiota, such as propionate and tryptophan, contribute substantially to radioprotection 184. Considering the correlation between the gut microbiota and radiation-induced gut damage, probiotics and prebiotics have been used in clinical interventions to prevent or treat radiation-induced gut injury 186,187. The results indicate the beneficial effects of the gut microbiota and its metabolites on radiation-induced gut damage; however, these findings are not yet sufficient to influence clinical practice 26. However, additional research is needed to confirm the protective effect of the gut microbiota and its metabolites on radiation-induced gut injury.

### 3.4 Molecular targeted therapy

Molecular targeted therapy has increasingly been utilized in the treatment of malignant tumors, establishing itself as a novel paradigm for tumor drug therapy. Compared to conventional therapies such as radiotherapy and chemotherapy, targeted therapy has superior efficacy and reduced toxicity 188. However, the adverse reactions induced by molecular targeted drugs cannot be disregarded. Diarrhea represents a prevalent clinical manifestation that not only compromises patient quality of life but also imposes limitations on the safe utilization of these drugs 189. Increasing evidence suggests that the gut microbiota could influence the development of tyrosine kinase inhibitor (TKI)-induced diarrhea 190. Pal et al. collected stool samples from patients with metastatic renal cell carcinoma who were receiving vascular endothelial growth factor (VEGF)-TKIs and evaluated the relationship between VEGF-TKI-related diarrhea and the gut microbiota. They discovered higher levels of *Bacteroides spp.* and lower levels of *Prevotella spp.* in patients with diarrhea 191. Alterations in the gut microbiota can be observed in patients who experience TKI-induced diarrhea, and regulating these changes may reduce the occurrence of these side effects. Ianiro et al. reported findings from a randomized clinical trial (ClinicalTrials.gov number: NCT04040712) of FMT for the treatment of diarrhea induced by TKIs in patients with metastatic renal cell carcinoma 192. In this study, twenty patients were randomly assigned to receive FMT from either healthy donors or placebo-treated FMT. These findings demonstrate that donor FMT exhibits superior efficacy to placebo FMT in the treatment of TKI-induced diarrhea; additionally, successful engraftment is observed in recipients receiving donor feces.

Histone deacetylase (HDAC) inhibitors not only induce cellular differentiation, apoptosis, autophagy, and cell cycle arrest but also modulate immune responses and inhibit angiogenesis in various hematologic malignancies and some solid tumors 193. Butyric acid, an SCFA, accelerates histone acetylation and participates in the apoptosis and proliferation of various cancer cells. It has been extensively investigated as an HDAC inhibitor in the field of antitumor research 194,195. He et al. revealed that butyrate, a metabolite of the gut microbiota, can enhance the immune response of CD8^+^ T cells in an ID2-dependent manner, thereby improving the effectiveness of oxaliplatin in antitumor therapy 25. Luu et al. also demonstrated that pentanoate and butyrate modulate CD8^+^ T-cell responses, enhancing the antitumor activity of cytotoxic T lymphocytes and CAR-T cells 168. Moreover, their research suggested that *M. massiliansis* may be a potential probiotic for the production of pentanoate and butyrate.

### 3.5 Surgical treatment

In the early stages of cancer, surgery is commonly used as a treatment method and significantly impacts patient microbiota, especially the gut microbiota 196. Research has shown that in patients undergoing tumor surgery, the use of preoperative antibiotics may lead to a reduction in gut microbial diversity and the growth of pathogenic bacteria, potentially resulting in complications such as increased gut permeability 197,198. Modulating the gut microbiota can be considered a potential strategy to alleviate this issue. Relevant studies have shown that certain microorganisms, such as *Lactobacillus spp.* and *A. muciniphila*, can regulate gut barrier healing through mechanisms dependent on ROS or formyl peptide receptors 199,200. The *Bacteroides thetaiotaomicron* is an important component of the gut microbiota in normal mice and humans. It has been demonstrated that these commensal bacteria can successfully colonize germ-free mice and significantly regulate the expression of genes associated with various gut functions, such as nutrient absorption, mucosal barrier reinforcement, and angiogenesis 201. In contrast to probiotics that preserve the integrity of the gut barrier, certain pathogenic bacteria, such as *Serratia marcescens* and *Pseudomonas aeruginosa*, can exacerbate damage to the gut barrier 202. Therefore, selectively eliminating pathogenic bacteria and preserving probiotics before surgery can effectively mitigate surgical complications. Currently, several ongoing studies are investigating the impact of perioperative probiotics and commensal bacteria on surgical complications in patients undergoing tumor resection 203.

Mounting evidence suggests that the gut microbiota plays a crucial role in modulating both the efficacy and toxicity of cancer therapy 30,148. However, the field is in its infancy, and tremendous opportunities exist to further elucidate the mechanisms through which these microorganisms impact cancer therapy. Therefore, it is crucial to explore a comprehensive approach that integrates the regulation of the gut microbiota with cancer immunotherapy, intensive chemotherapy, radiotherapy, targeted therapy, and surgery to achieve enhanced therapeutic outcomes while minimizing adverse effects. Currently, numerous ongoing clinical trials are underway to translate research findings from laboratory experiments to practical applications 147. Moreover, considering the substantial variation in bacterial strains among different healthy individuals and the limited functional understanding of the gut microbiota, coupled with a lack of knowledge regarding the composition of an “optimal” bacterial consortium, caution should be exercised when regulating the gut microbiota in cancer patients.

## 4. The gut microbiota: A new force in cancer diagnosis?

An increasing number of animal experiments and clinical studies have demonstrated that the diversity and abundance of the gut microbiota are associated with cancer pathogenesis and treatment 10,17. These data illustrate the potential use of the gut microbiota as a biomarker for understanding cancer pathogenesis and guiding cancer treatment 18,132,204.

### 4.1 Screening for cancer

The utilization of the gut microbiota as a biomarker for cancer diagnosis is being extensively studied in both preclinical and clinical research 205. Timely treatment for early-stage cancer can lead to effective therapeutic outcomes. For example, the 5-year survival rate for patients with localized CRC is 90%, while that for patients with distal metastatic CRC is only 14% 16. Many HCC patients are already in the advanced stage at the time of diagnosis, and the gut microbiota could serve as a reliable biomarker for the early screening of HCC 132,133. Researchers collected fecal samples from individuals in East China, Central China, and Northwest China and analyzed the fecal microbiota using HTST. These feces were obtained from healthy individuals, patients with liver cirrhosis, and patients in the early stage of liver cancer. Screening with a random forest model revealed that 30 gut microbial markers can most accurately reflect the progression of liver cancer; the area under the curve (AUC) was 0.8. The model was validated in liver cancer patients from Northwest and Central China, and the AUC for differentiating between healthy individuals and those with early-stage liver cancer was 0.768, while it was 0.804 for differentiating between healthy individuals and those with advanced-stage liver cancer 132. This model establishes a connection between changes in the gut microbiota and liver cancer screening, emphasizing the potential of the gut microbiota as a diagnostic tool for liver cancer.

Given that gut dysbiosis is typically an early event in the development of CRC, numerous studies have been conducted to explore the fecal microbiome to identify potential diagnostic markers 205,206. Kong et al. conducted metagenomic and metabolomic analyses of the interactions among the gut microbiota, metabolites and microbial enzymes in 130 individuals with late-onset CRC (LO-CRC), 114 individuals with early-onset colorectal cancer (EO-CRC), and age-matched healthy controls to assess the potential of those factors to serve as noninvasive biomarkers for EO-CRC 97. Compared to that in the control group, the alpha diversity in both the LO-CRC and EO-CRC groups was lower. The enrichment of *F. nucleatum* and depletion of SCFAs are characteristic features observed in LO-CRC. In comparison, the multiomics signatures of EO-CRC exhibited a tendency toward an increased presence of *Flavonifractor plauti* and elevated levels of tryptophan, bile acid and choline metabolism. Yu et al performed HTST on a cohort of CRC patients (n=74) and healthy individuals (n=54) and reported that 20 types of gut microbiota were associated with CRC; the AUC was 0.84 204.

### 4.2 Predictive biomarkers

The available data suggest a correlation between specific gut bacteria and cancer prognosis, indicating that microbial markers have the potential to predict the treatment response of cancer patients. However, there is a paucity of data from studies that specifically investigate the longitudinal changes in the gut microbiota during chemotherapy, radiotherapy, and molecular targeted therapy. Given the pivotal role of the gut microbiota in facilitating an efficacious response to ICIs, numerous studies have endeavored to establish correlations between specific gut microbial signatures and ICI responses and survival outcomes. Associations between specific bacterial species, such as *A. muciniphila* 162,165 and *Bifidobacterium spp.* 28, and the response to ICIs have been extensively documented. Martini et al. compared responders and nonresponders in a cohort of 14 patients with CRC who received cetuximab plus the antiPDL1 antibody avelumab 207. They found that, compared to nine patients with shorter progression-free survival (PFS) (2-6 months), five long-term responding patients (those with PFS 9-24 months) had significantly greater abundances of two butyrate-producing bacteria, *Agathobacter M104/1* and *Blautia SR1/5*. These findings were validated in the CAVE-Lung validation cohort. In addition, improved predictors of ICI response are essential for optimizing the efficacy of this therapeutic approach.

The administration of antibiotics compromises the therapeutic efficacy of PD-1 blockade in cancer patients 163; antibiotic treatment leads to a decrease in gut microbiota diversity and an increase in the abundance of *Enterocloster clostridioformis*, which subsequently downregulates the serum mucosal addressin cell adhesion molecule-1 (MAdCAM-1) level. A low serum concentration of MAdCAM-1 has a negative impact on prognosis, which has been verified in a cohort of NSCLC patients 163,208. Therefore, it is imperative to investigate the impact of antibiotic exposure on the outcome of ICI treatment.

Treatment with combined immune checkpoint blockade (CICB) agents targeting both CTLA-4 and PD-1 has demonstrated remarkable clinical efficacy across various tumor types; however, this efficacy comes at the cost of frequent, severe immune-related adverse events 209. Andrews et al. demonstrated that there is a correlation between gut microbiota signatures and the toxicity associated with CICB 210. Gut microbiota profiling revealed a significantly greater abundance of *Bacteroides intestinalis* in patients experiencing toxicity than in patients without toxicity, and the gut microbiota mediated CICB-induced intestinal toxicity through IL-1β; however, the underlying mechanism requires further elucidation.

### 4.3 Future directions

**Figure 6:**
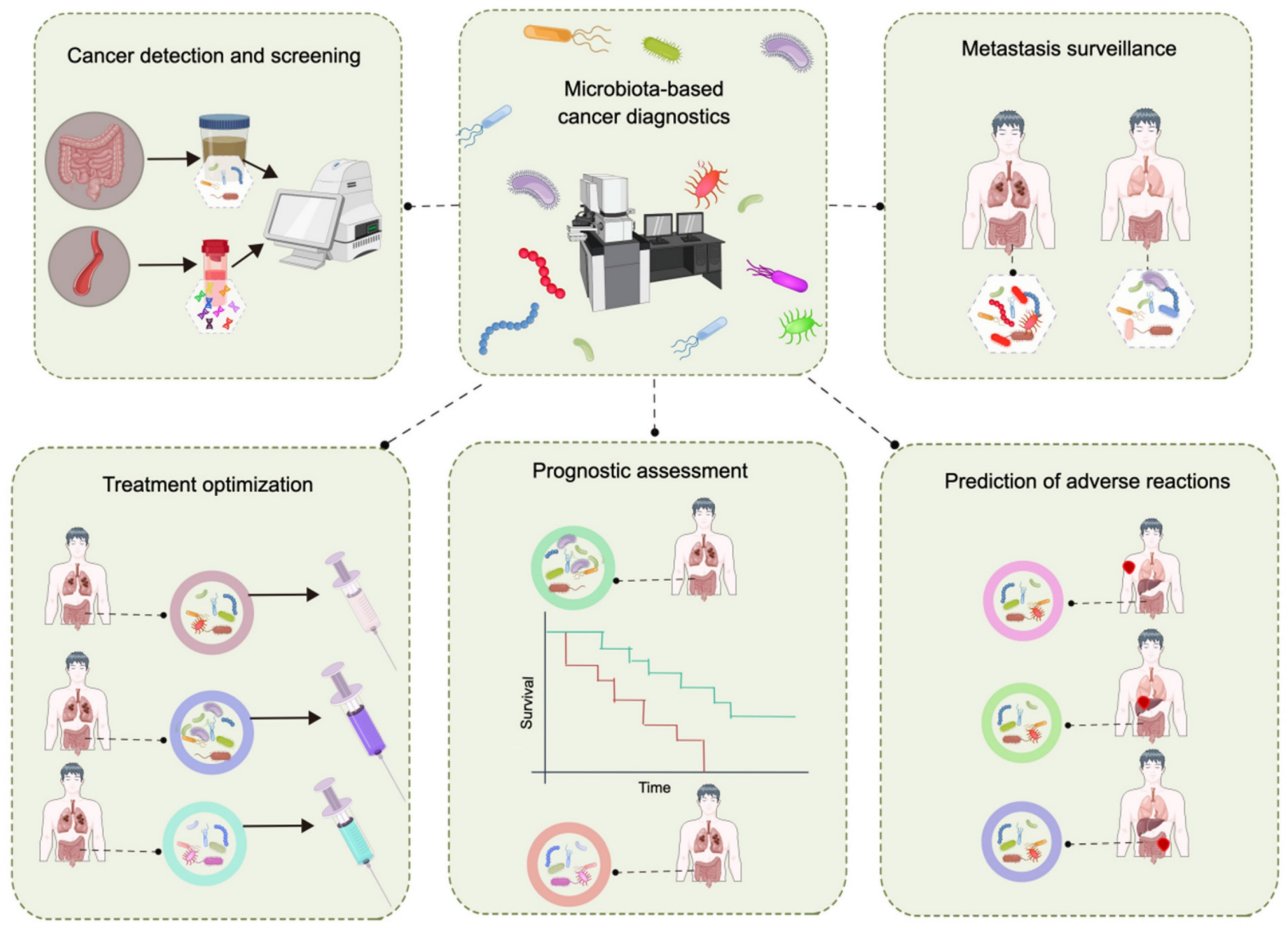
Utilization of gut microbiota data in cancer diagnosis and patient stratification. This figure was created using Figdraw.

Extensive research has confirmed the relationship between the gut microbiota and cancer. As research continues to elucidate the corresponding mechanisms, unprecedented opportunities arise for exploring the use of the gut microbiota in cancer diagnosis and management; however, challenges remain. The primary limitation lies in the accuracy of utilizing fecal microbial markers for cancer screening 77,211. To address this issue, the integration of the gut microbiota with other biomarkers has been employed to increase the precision of detection. Numerous studies have demonstrated the close relationship between *F. nucleatum* and the initiation of CRC. The combination of fecal immunochemical detection with the abundance of *F. nucleatum* significantly enhances the efficacy and sensitivity of fecal immunochemical detection 212,213. The fecal immunochemical test has an AUC value of 0.86 for CRC detection; however, incorporating the abundance of *F. nucleatum* into the model further increases the AUC value to 0.95 214. In addition, the integration of the gut microbiota and diagnostic biomarkers in serum enhances the precision of cancer detection via the gut microbiota. The fecal metagenomic classifier had an AUC of 0.84 for accurately identifying pancreatic ductal adenocarcinoma (PDAC) within a Spanish cohort, and the accuracy improved (AUC of 0.94) when combined with the less specific carbohydrate antigen (CA) 19-9 serum marker 215. CA19-9 is currently the only FDA-approved noninvasive diagnostic biomarker for PDAC and has a low specificity for diagnosing PDAC 215,216. The incorporation of the gut microbiota and other biomarkers enhances the precision of cancer detection. In future investigations, evaluating the combined utilization of the gut microbiota and additional biomarkers will be particularly crucial. The ultimate goal is to develop a method based on the gut microbiota for early cancer detection, metastasis surveillance, treatment optimization, etc. (Figure 6).

## 5. Modulating the gut microbiota

**Figure 7:**
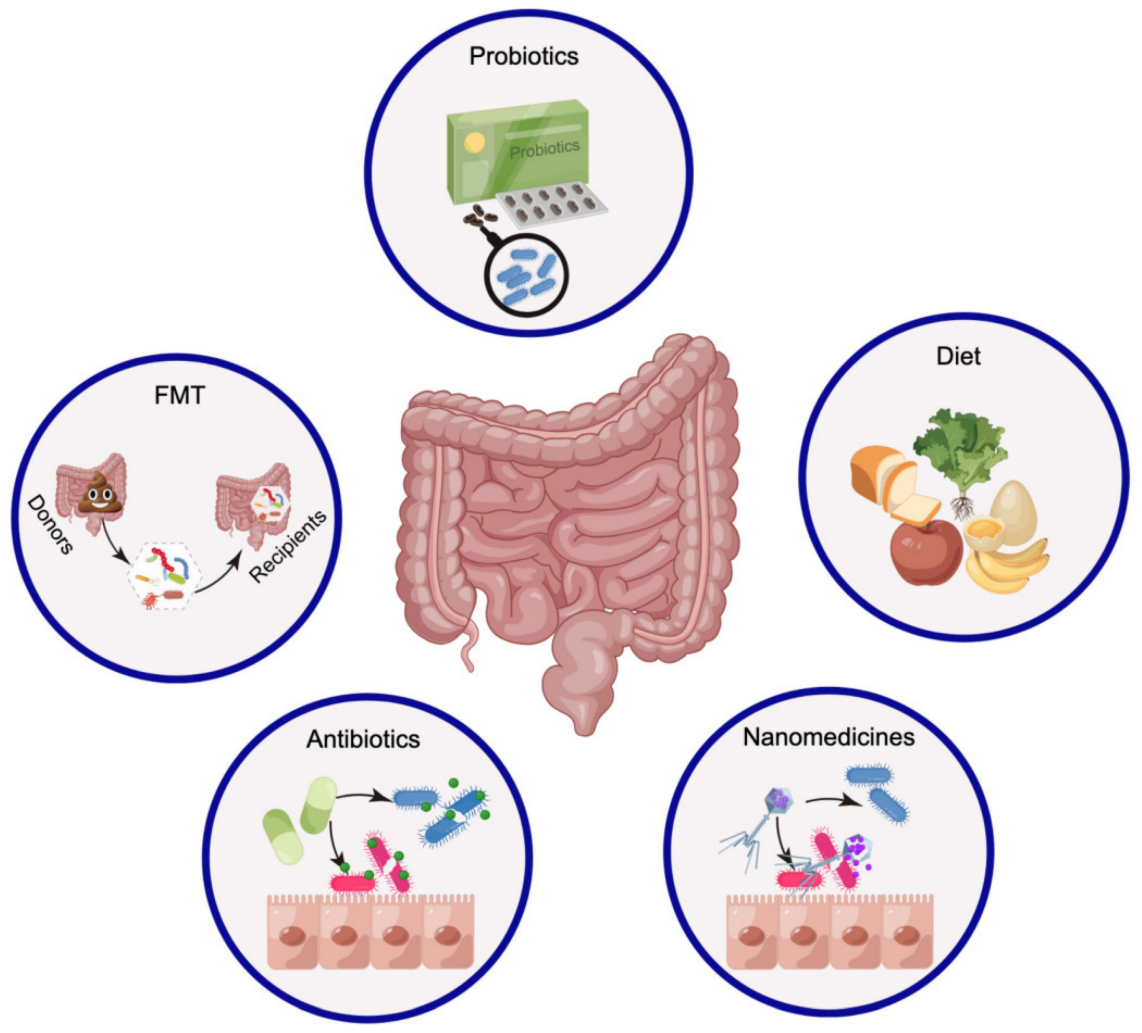
Strategies to modify the gut microbiota for cancer treatment The modulation of the gut microbiota through FMT, probiotics, and dietary regulation primarily contributes to the enrichment of probiotics. The current practice primarily involves the use of antibiotics for eradicating pathogenic bacteria, which may have detrimental effects on treatment outcomes. However, the application of nanomedicines offers opportunities for targeted elimination of pathogenic bacteria. This figure was created using Figdraw.

Numerous studies have shown that cancer- and host-related factors combine in different ways, revealing the heterogeneity of cancer pathogenesis and clinical treatment outcomes. How can the gut microbiota be utilized for cancer treatment? Modulating the gut microbiota may be a manipulable and beneficial approach to cancer treatment when considering all factors 52,217. Although people hope to improve the efficacy of tumor treatment by modulating the gut microbiota, there is currently a lack of consensus on how to regulate this process. Currently, FMT 217, probiotics 26, dietary adjustments 218, and antibiotics 219 are utilized primarily for modifying the composition of the gut microbiota (Figure 7). Notably, nanomedicine is prepared in an interdisciplinary manner with the aim of targeting and eliminating specific pathogenic bacteria 38.

### 5.1 Fecal microbiota transplantation

FMT has been studied in the context of cancer treatment, and restoring the recipient's gut microbiota to an optimal health status is the most direct method; this approach poses both a major challenge and an urgent opportunity 217. FMT preparations can be administered through oral delivery via freeze-dried or frozen pellets, as well as invasive procedures such as colonoscopy or gastroscopy 26. FMT transplants a complete gut microbiota from the donor to the recipient, and the introduced microbiota is more stable in the recipient's environment and less competitive against the recipient's own microbiota, thereby facilitating microbial interdependence and collaboration 203. FMT has achieved excellent results in the treatment of *Clostridium difficile* infections, demonstrating a greater cure rate than standard therapy 203. Moreover, FMT is considered a viable treatment option for various diseases, including diabetes, metabolic syndrome, and inflammatory bowel disease 220,221. Currently, clinical trials of ICI therapy involving FMT are underway. A study by Routy et al. demonstrated that FMT enhances the effectiveness of anti-PD-1 therapy 37.

While research on FMT is flourishing, a patient in the U.S. died after receiving FMT in 2019, and as a result, the U.S. FDA suspended some clinical trials involving FMT until safety was fully confirmed. The poor efficacy of FMT raises concerns regarding the potential risk of infection 222. The common adverse effects of FMT often pertain to gastrointestinal discomfort, which can include abdominal cramps, constipation, bloating, hiccups, nausea, vomiting, diarrhea, hematochezia and so on. However, these symptoms usually resolve quickly and do not pose a significant threat to patient health 223. The improper screening of donors and inadequate analysis of fecal donor material may also result in serious adverse reactions. For example, donor feces were not screened, and as a result, two patients contracted multidrug-resistant bacteria after FMT, leading to one death. Therefore, the FDA has warned researchers to expand fecal screening in FMT studies to include specific antibiotic-resistant bacteria. However, this measure alone is insufficient for predicting adverse events caused by specific pathogen infections. These infections may not contain antibiotic-resistant bacteria, but pathogenic bacteria derived from the donor could still possess inherent virulence and pose a threat to recipients' health 222. Conducting thorough screening tests on FMT donors is essential for reducing and preventing the incidence of adverse events 221,222.

Additionally, successful FMT requires not only the transplantation of microbiota into the recipient's gut but also long-term colonization to maintain therapeutic efficacy 224,225. After FMT, the gut microbiota of the recipient and donor exhibit the highest similarity on the first day, but the composition changes over time 226. The average duration for maintaining a clinical response in patients with Crohn's disease is 125 days after the initial FMT and 176.5 days after the second transplantation 227. These findings suggest that FMT can be regularly performed to maintain clinical efficacy. Currently, there are limited clinical studies on the application of FMT as an adjuvant cancer treatment; however, ensuring its safety and long-term efficacy are important concerns.

### 5.2 Probiotics

Although FMT is the most direct method for altering the gut microbiota, the complex microbial community increases the risk of infection in patients 222,223. Compared to FMT, probiotic transplantation provides a more practical approach for regulating the gut microbiota in clinical treatment 26. The term “probiotics” refers to live microorganisms that, when administered in appropriate quantities, provide a safe beneficial effect on the host's health 228. The earliest commercial probiotic supplements were derived from easily cultivable single strains of food sources, such as *Bifidobacterium* and *Lactobacillus*, which have well-established uses in the treatment of numerous gastrointestinal disorders 229,230. Given such observations, is it feasible to utilize probiotics in cancer treatment?

Research on the use of probiotics for cancer treatment has focused mainly on their ability to enhance immune function, potentially helping combat cancer 203. In CRC patients treated with *Lactobacillus johnsonii* during the perioperative period, bacteria adhere to the colonic mucosa, reducing the concentration of pathogens in feces and regulating local immune function 231,232. Additionally, *A. muciniphila* is capable of restoring mouse responsiveness to PD-1 inhibitors 165. *Bifidobacterium* has shown potential in enhancing antitumor immunity and improving the effectiveness of anti-PD-L1 treatments 28. The administration of probiotics enhances the immune response and mitigates the adverse effects of radiation therapy. Treatment with *Lactobacillus acidophilus LAC-361* and *Bifidobacterium longum BB-536* can reduce radiation-induced diarrhea 233. The aforementioned cases exemplify the promising potential of probiotics in the field of cancer therapy.

Despite the demonstrated benefits of probiotics in cancer treatment, there are still numerous challenges that need to be addressed. Probiotics vary in their ability to survive gastric acid treatment and colonize the gut, just as their species, dosage, preparation method, and host microbiota also differ 228. The vast majority of clinical trials on probiotics reported in the literature have not raised significant safety concerns; however, there are still some serious adverse reactions caused by probiotics that draw our attention to their potential risks. These reported cases involve incidents of bacterial sepsis associated with *Lactobacilli*-containing probiotic supplements, as well as the death of a preterm infant from gastrointestinal mucormycosis associated with mold contamination in a probiotic supplement 234. In addition, probiotics are used to regulate the gut microbiota but are largely unregulated in both the EU and the US, potentially resulting in significant variations in quality 234. Currently, there are no universally applicable probiotics available for modulating the gut microbiota. Prior to administering probiotics to cancer patients, individual analysis and cautious usage are warranted, with tailored strategies developed for specific populations 26. Strategies for selecting probiotics should be considered. The safety assessment of probiotics is of paramount importance. In what combinations can probiotics be used (such as joint synbiotics), what is the timing of use, and what are the mechanisms of action 235? How can successful microbial treatments be efficiently packaged, deployed, and dosed over time to achieve effective treatment or reduce treatment side effects?

### 5.3 Diet

Considering the crucial role of the gut microbiota in preventing cancer and the limitations associated with FMT and probiotics, most researchers have incorporated diet into their studies on regulating the gut microbiota. Diet plays a crucial role in determining the structure and function of the gut microbiota, and the interaction between diet and microorganisms determines whether they are beneficial or detrimental to host health 218,236. Considering a series of parameters, the Mediterranean diet is associated with a lower risk of cancer initiation and death, primarily by enhancing immune function mediated by cytotoxic cells and helper T cells 237. Is it possible to modulate the gut microbiota in a way that is beneficial to human health through specific dietary components?

Analyses of dietary components have revealed that certain ingredients can influence the composition and abundance of specific gut microbiota 203,236. The supplementation of dietary fibers, such as fructan and galactooligosaccharide, alters the composition of the gut microbiota, increases the abundance of *Bifidobacterium* and *Lactobacillus spp.*, subsequently increases the concentration of butyrate in feces, and inhibits CRC 238-240. Resistant starch is a substance that benefits gut health by serving as a valuable substrate for numerous beneficial gut microorganisms, including the genera Bifidobacterium, *Akkermansia*, and *Megasphaera* 241,242. The combination of resistant starch with arabinoxylan increases the abundance of *Bifidobacterium* while decreasing the abundance of other undesirable genera in the gut microbiota, thus modulating the concentration of SCFAs in the gut and exerting beneficial effects on colon health 243. The results of that study suggest, to some extent, that the use of resistant starch and arabinoxylan for modulating the gut microbiota may enhance the therapeutic effect of CRC treatment. Preclinical and clinical studies have demonstrated that both the type and quantity of protein in the diet impact the composition of the gut microbiota 218. Studies have shown that casein acts as a growth factor for *Lactobacilli* and *Bifidobacteria*; additionally, these bacteria have been shown to be beneficial in cancer treatment 218.

### 5.4 Elimination of pathogenic microorganisms

Research indicates that the gut microbiota plays a significant role in the initiation, progression and treatment of cancers such as BC 44, CRC 78, and HCC 129,131. The main way to regulate the gut microbiota mentioned above is by increasing the abundance of beneficial bacteria, aiming to prevent cancer initiation and aid in cancer treatment. However, eradicating pathogenic microorganisms is equally crucial for both cancer prevention and treatment. Antibiotic treatment is the most common method for eliminating pathogenic microorganisms, but this indiscriminate elimination can harm probiotics 219,244. The targeted elimination of pathogenic microorganisms through nanomedicine offers greater possibilities for effectively regulating the gut microbiota 38.

#### 5.4.1 Antibiotics

The use of antibiotics in the treatment of diseases is becoming increasingly prevalent among humans. The scavenging effects of antibiotics on the microbiota are well known; they can eliminate pathogenic microorganisms but may also disrupt the structure of the microbial community in the human body, leading to the dysregulation of host-microbiota interactions 219,244. Even at sublethal concentrations, antibiotics can cause significant and nonselective changes in the gut microbiota. Furthermore, Parthasarathy et al. reported that the impact of antibiotics on slow-growing and aggregating microorganisms is more pronounced than that on rapidly growing microorganisms 245. A study conducted by Hagan et al. demonstrated that antibiotic usage led to a 10,000-fold decrease in the gut microbiota load 58. Additionally, both the diversity and abundance of the gut microbiota decreased, and the bacteria failed to fully recover within six months. These findings suggested that certain specific microbial species may be absent for a prolonged period following antibiotic use. They also found that antibiotics not only affect the composition of the gut microbiota but also disrupt blood metabolism, such as bile acid and tryptophan metabolism. The nonselective eradication of the gut microbiota by antibiotics compromises the therapeutic efficacy of PD-1 blockade in cancer patients 162. Fidelle et al. reported that antibiotic treatment leads to a decrease in gut microbiota diversity and an increase in the abundance of *Enterocloster clostridioformis*, resulting in a low serum soluble MAdCAM-1 level and thus a negative impact on prognosis 163. Therefore, it is particularly important to selectively eliminate pathogenic microorganisms to minimize this impact.

#### 5.4.2 Nanomedicines

Nanomaterials can serve as carriers for delivering various therapeutic drugs to target sites, thereby prolonging their circulation time, protecting drugs from degradation, and reducing drug accumulation at nontarget sites to minimize side effects 246-248. Utilizing these advantages of nanomaterials to prepare nanomedicines makes it possible to selectively eliminate pathogenic microorganisms. The targeting effect of phages is noteworthy for the specific eradication of pathogenic microorganisms 249,250. Inspired by this, Zhang et al. designed a targeted nanomedicine to eradicate *F. nucleatum*, as multiple studies have shown its association with the initiation and progression of CRC 38. They isolated a phage that specifically lysed *F. nucleatum*, which was subsequently modified with azide. Dextran nanoparticles were used to coat anti-CRC drugs, and these drugs were covalently linked to the azide-modified phages. These phage-mediated nanoparticles can target *F. nucleatum* without binding to *Bacillus thuringiensis*, *E. coli*, or *Clostridium butyricum*. This bacteriophage-mediated nanomedicine specifically targets and modulates the composition of the gut microbiota, thereby enhancing the therapeutic efficacy of chemotherapy for CRC. In another study, the capsid protein of this phage was electrostatically assembled with silver nanoparticles to achieve specific clearance of *F. nucleatum* in the gut and reshape the tumor immune microenvironment, significantly extending overall survival in CRC mice 89. These studies demonstrate the potential of utilizing nanomaterials to selectively eliminate pathogenic microorganisms, which could be an effective therapeutic strategy for modulating the gut microbiota in the future.

The era of the gut microbiota is ongoing, and the role of the gut microbiota in cancer therapy has been extensively reported in preclinical and clinical studies, suggesting that the gut microbiota may become a potential factor in cancer treatment 26. However, investigations of the impact of modulating the gut microbiota on cancer treatment have relied primarily on murine models, with few clinical trials being carried out 203. The human gut microbiota differs significantly from that of mice; therefore, safety assessments should be conducted before extrapolating results from mouse trials to humans 251.

## 6. Summary and perspectives

The gut microbiota, referred to as the “second genome” of the human body, plays an undeniable role in human health; however, it is also closely associated with various diseases. There is substantial evidence suggesting that the gut microbiota is associated with the initiation of CRC, HCC, BC, and other types of cancer. Recent research on the role of pathogenic microorganisms in cancer has focused primarily on determining the correlation between the abundance of specific strains and cancer using HTST and elucidating the underlying mechanisms that contribute to tumorigenesis. Eliminating carcinogenic microorganisms can prevent cancer or benefit patients during cancer treatment. However, there are still many unresolved issues, such as how to remove pathogenic microorganisms without affecting other probiotics. The scavenging effects of antibiotics on the microbiota are widely acknowledged; however, their administration for eradicating the gut microbiota may inadvertently compromise probiotic populations, potentially leading to unintended consequences. The targeted elimination of detrimental gut microbiota constituents through nanomedicine represents a highly promising method for future exploration and necessitates further comprehensive investigation.

While certain gut microorganisms may be associated with the initiation of cancer, importantly, there are numerous beneficial gut microorganisms that play crucial roles in the body's defense against cancer. This includes enhancing the effectiveness of ICIs and chemotherapy, as well as reducing gut damage caused by chemotherapy and radiation therapy. Considering the advantageous characteristics of the gut microbiota, modulating the gut microbiota is expected to enhance the effectiveness of anticancer therapies. The current primary approaches employed include FMT, probiotic administration, and dietary interventions. The application of these methods can increase the abundance of probiotics, thereby strengthening the effectiveness of cancer treatment; however, they may also induce certain adverse reactions. The future holds promise for enhancing the efficacy of cancer treatment through personalized modulation of the gut microbiota through the use of appropriate interventions while minimizing intolerable adverse reactions.

Several researchers have proposed listing the gut microbiota as a biomarker for the diagnosis and management of cancer based on its impact on cancer pathogenesis and treatment. The sensitivity of the gut microbiota alone as a marker for cancer diagnosis and management may not be high; however, when combined with other markers, the detection sensitivity of the gut microbiota is significantly enhanced. The future holds promise for utilizing the gut microbiota as a noninvasive approach for cancer detection and assessment of treatment efficacy.

## Abbreviations

FMT: fecal microbiota transplantation
HTST: high-throughput sequencing technology
CRC: colorectal cancer
SCFAs: short-chain fatty acids
ICIs: immune-checkpoint inhibitors
BC: breast cancer
LC: lung cancer
UGT: uridine 5′-diphospho-glucuronosyltransferase
gmGUS: gut microbial β-glucuronidase
TLR: toll-like receptors
NSCLC: non-small cell lung cancer
SCLC: small cell lung cancer
rRNA: ribosomal RNA
MDSCs: myeloid-derived suppressor cells
TAM: tumor-associated macrophages
TANs: tumor-associated neutrophils
PRR: pattern recognition receptor
H2S: hydrogen sulfide
PCa: prostate cancer
ADT: androgen deprivation therapy
CRPCa: castration-resistant prostate cancer
CT: surgical castration
CS: castration-sensitive phase
CR: castration-resistant phase
GC: gastric cancer
NO: nitric oxide
HCC: hepatocellular carcinoma
LPS: lipopolysaccharide
FDA: Food and Drug Administration
PAMPs: pathogen-associated molecular patterns
DCs: dendritic cells
CAR: chimeric antigen receptor
CpG-ODN: CpG-oligodeoxynucleotide
3-IAA: indole-3-acetic acid
ROS: reactive oxygen species
CTX: Cyclophosphamide
AUC: area under the curve
LO-CRC: late-onset CRC
EO-CRC: early-onset colorectal cancer
PFS: progression-free survival
MAdCAM-1: mucosal addressin cell adhesion molecule-1
CICB: combined immune checkpoint blockade
PDAC: pancreatic ductal adenocarcinoma
CA: carbohydrate antigen
